# Fatty acid synthase regulates invasion and metastasis of colorectal cancer via Wnt signaling pathway

**DOI:** 10.1002/cam4.711

**Published:** 2016-05-03

**Authors:** Haiyu Wang, Qiulei Xi, Guohao Wu

**Affiliations:** ^1^ Department of general surgeryZhongshan hospitalFudan universityShanghaiChina

**Keywords:** Colorectal carcinoma, fatty acid synthase, metastasis, Wnt signaling

## Abstract

Fatty acid synthase (Fasn) is the key metabolic enzyme that accounts for the terminal catalytic step in fatty acid synthesis, which is hyperactivated in various tumors. In this study, we depicted that Fasn expression was elevated in human colorectal cancer (CRC), which accordingly led to lymphatic and distant metastasis and a more advanced clinical phenotype. Genetic perturbations demonstrated that knocking down Fasn inhibited cell migration and invasion both in SW480 and HT29 CRC cell lines. Further mechanical exploration revealed that Fasn knockdown could attenuate Wnt signaling pathway via downregulating distinctive genes, namely Wnt5a, Wnt5b, Fzd2, which at least partly contributed to the decrease in metastasis. Clinical evidence verified a positive correlation between Fasn expression and Wnt signal marker gene expression in a cohort of 43 CRC patients. In conclusion, we shed light on metabolic switches took place during CRC carcinogenesis, among which Fasn is a critical factor and a potential therapeutic target.

## Introduction

Colorectal carcinoma (CRC) is the third most common malignant disease in men and the second in women 1 worldwide and its prevalence and mortality continued to increase in the Asia‐Pacific region. Despite the development of new treatment in last decade 2, surgery resection is still considered the optimal treatment for CRC patients. Although neoadjuvant treatments constitute a crucial part of an integrated antitumor therapy, stacking studies have shown that high financial cost 3, resistance, and toxicity severely constrained the effectiveness of the treatment, which have been the largest impediment all along. Therefore, it is essential to identify biomarkers of advanced CRC and set up new therapeutic strategy for clinical treatments 4.

It has come to the senses of many scientists that metabolic reprogramming is an emerging hallmark during CRC 5. Among all the metabolic alterations, enhanced lipogenesis is particularly important 6. This de novo lipogenic conversion starts early when cells first acquire cancerous phenotype and further extends as the tumor cells become more aggressive, suggesting that upregulation of fatty acid synthesis is indispensable during carcinogenesis in order to fuel cell proliferation and to build membrane phospholipids. Fatty acid synthase (Fasn) is a key lipogenic enzyme catalyzing the terminal steps in the de novo biogenesis of fatty acids 7, which were underused in normal cells and were unexpectedly massively consumed in various tumors and their precursor lesions. Although several studies have delineate oncogenic nature of Fasn‐driven lipogenesis, the function of Fasn in CRC patients is discrepant due to change of region or population and the precise mechanisms underlying remain equivocal 8.

In this study, we identified varied but collectively elevated expression of Fasn in colorectal carcinoma. Utilizing shRNA interference to analyze the possible function of Fasn during CRC carcinogenesis, we were able to detect that Fasn played critical role in CRC cell invasion mainly through activating Wnt signal pathway. Further assessment revealed a correlation between Fasn expression level and a more malignant clinical phenotype in regard to distant metastasis, advanced stage, and hence poor prognosis. In summary, we demonstrated the oncogenic nature of Fasn in CRC leastways through driving Wnt‐dependent invasion. Since Fasn inhibitor is widely accessible, Fasn not only could serve as an oncogenic biomarker, but also offer new therapeutic opportunities for metabolically abnormal late‐stage and metastatic CRC patients.

## Materials and Methods

### Cell culture

Coloreatal cancer cell line HT‐29, SW480, SW620, and SW1116, and embryonic kidney cell line HEK293T were purchased from Cell Bank of Chinese Academy of Sciences. HT‐29 cells were cultured in Dulbecco's modified Eagle's medium (DMEM) supplemented with 10% fetal bovine serum (FBS), penicillin (100 U/mL), and streptomycin (100 *μ*g/mL), and were incubated at 37°C in a humidified incubator under 5% CO_2_ condition.

SW480, SW620, and SW1116 cells were cultured in L‐15 medium (DMEM) supplemented with 10% fetal bovine serum (FBS), penicillin (100 U/mL), and streptomycin (100 *μ*g/mL), and were incubated at 37°C in a humidified incubator under 0.038% CO_2_ condition.

### Immunohistochemical staining

Paraffin‐embedded CRC and adjacent normal tissue specimens were obtained from the Division of General Surgery at Zhongshan hospital. Formalin‐fixed paraffin‐embedded (FFPE) 6 mm sections were used for immunohistochemical (IHC) analysis. Fasn primary antibody (proteintech, cat# 10624‐2‐AP, 1:50 dilution) was incubated overnight at 4°C. Immunostaining was performed using diaminobenzidine reaction and controlled under microscope. Slides were counterstained with hematoxylin.

All IHC staining was assessed independently by two pathologists and scored according to the ratio and intensity of positive staining. Briefly, the ratio was graded from 1 to 4 based on the percentage of positive staining cells (1, 0–5%; 2, 6–50%; 3, 51–75%; 4, 76–100%). The intensity was graded from 1 to 4 (1, no staining; 2, weak staining; 3, moderate staining; 4, strong staining). A final score from 1 to 16 was calculated by multiplying ratio and intensity score. For each sample, it was indicated as negative (1–4), weakly positive (5–8), moderately positive (9–12), or strongly positive (>12).

### Quantitative real‐time PCR

Total RNA was extracted from cultured cells using Trizol reagent (Invitrogen, Carlsbad, CA) and dissolved in diethylpyrocarbonate treated (DEPC) water. cDNA was synthesized using the Takara Reverse Transcription System Kit (Takara Biotechnology Co. Ltd., Kusatsu, Japan) according to the manufacturer's instruction. Real‐time quantitative RT‐PCR was performed using the Sybr green premix kit (BioRad, Hercules, FL). All reactions were done in triplicates. GAPDH was used as a housekeeping gene. The following primers are used in this assay: Fasn—sense AAGGACCTGTCTAGGTTTGATGC and antisense TGGCTTCATAGGTGACTTCCA; Wnt5a—sense ATTCTTGGTGGTCGCTAGGTA and antisense CGCCTTCTCCGATGTACTGC; Wnt5b—sense CGCTTCGCCAAGGAGTTTG and antisense TGCCATCTTATACACAGCCCT; Fzd2—sense GTGCCATCCTATCTCAGCTACA and antisense CTGCATGTCTACCAAGTACGTG; GAPDH—sense ACAACTTTGGTATCGTGGAAGG and antisense—GCCATCACGCCACAGTTTC.

### Fasn shRNA transfection

Fasn shRNAs were obtained from Institute of Biochemistry and Cell Biology, SIBS, CAS. A scrambled sequence was used as a control. Cell transfection was performed with PEI reagent (Sigma‐Aldrich, St. Louis, MO).

### Matrigel invasion assay

For transwell invasion assay, 50 *μ*L matrigel (BD Bioscience, Franklin Lakes, NJ) was added into top chamber for 30 min at 37°C. Cells (1 × 104 cells/well) was starved in serum‐free medium for 24 h and plated to the top chambers. The bottom chambers were filled with completed medium. Any noninvading cells remaining in the top chamber were removed carefully in 48 h culturing. After fixed in methanol and stained with crystal violet, cells adhering to the lower membrane of the well were counted and imaged under ×200 magnification. Crystal violet staining was dissolved in 33% acetic acid and optical density was detected at 570 nm. The assay was carried out three times.

### Western blotting

Equal amounts of protein were separated by 10% SDS polyacrylamide gel electrophoresis and transferred to PVDF membranes. After blocking with 5% nonfat milk in TBST for 1 h, membranes were incubated overnight at 4°C with Fasn, GAPDH, Wnt5a, Wnt5b, and Fzd2 primary antibodies followed by incubation with HRP‐conjugated secondary antibodies. Immunoreactive bands were detected with enhanced chemiluminescent HRP substrate (Millipore, Bollerica, MA).

### Statistical analysis

Statistical analyses were performed using SPSS 21.0 (IBM, Chicago, IL). Relations between clinical parameters and Fasn expression levels were analyzed using chi‐squared test. In vitro studies were evaluated with ANOVA *t* test. Overall survival rate was estimated by Kaplan–Meier method. *P* < 0.05 was considered statistically significant.

## Results

### Fasn is overexpressed in CRC cell lines

**Figure 1:**
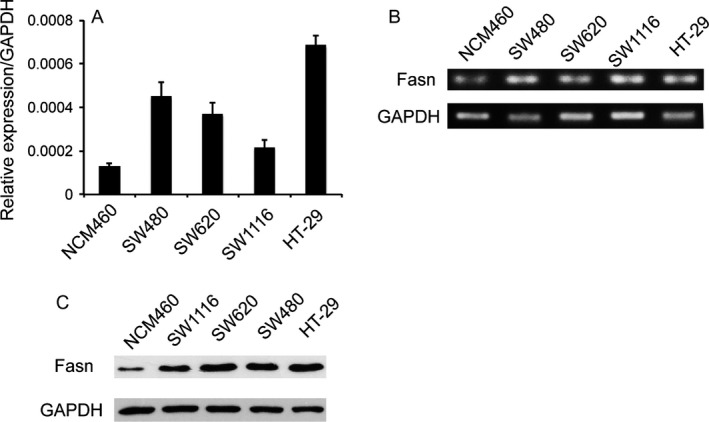
Fasn is overexpressed in CRC cell lines. (A) Quantitative RT‐PCR analysis of Fasn expression in four CRC cell lines and non‐CRC 293FT cell line. (B) Semiquantitative RT‐PCR analysis of Fasn expression in four CRC cell lines and non‐CRC 293FT cell line. (C) Western blotting analysis of Fasn protein expression in four CRC cell lines and non‐CRC 293FT cell line.

Fasn is a key enzyme catalyzing terminal step in fatty acid synthesis, which is widely expressed across versatile cell types 9. We examined Fasn mRNA and protein level in four CRC cell lines and non‐CRC NCM460 cell. As shown in Figure 1A and B, both quantitative and semiquantitative PCR displayed higher Fasn mRNA level in all four CRC cell lines (SW480, SW620, SW1116, and HT‐29), compared to non‐CRC NCM460 cells. Figure 1C confirmed that Fasn protein level is also relatively higher in CRC cell lines, among which SW480 and HT‐29 cells showed significantly higher Fasn expression.

### Fasn is overexpressed in the samples of CRC patients

**Figure 2:**
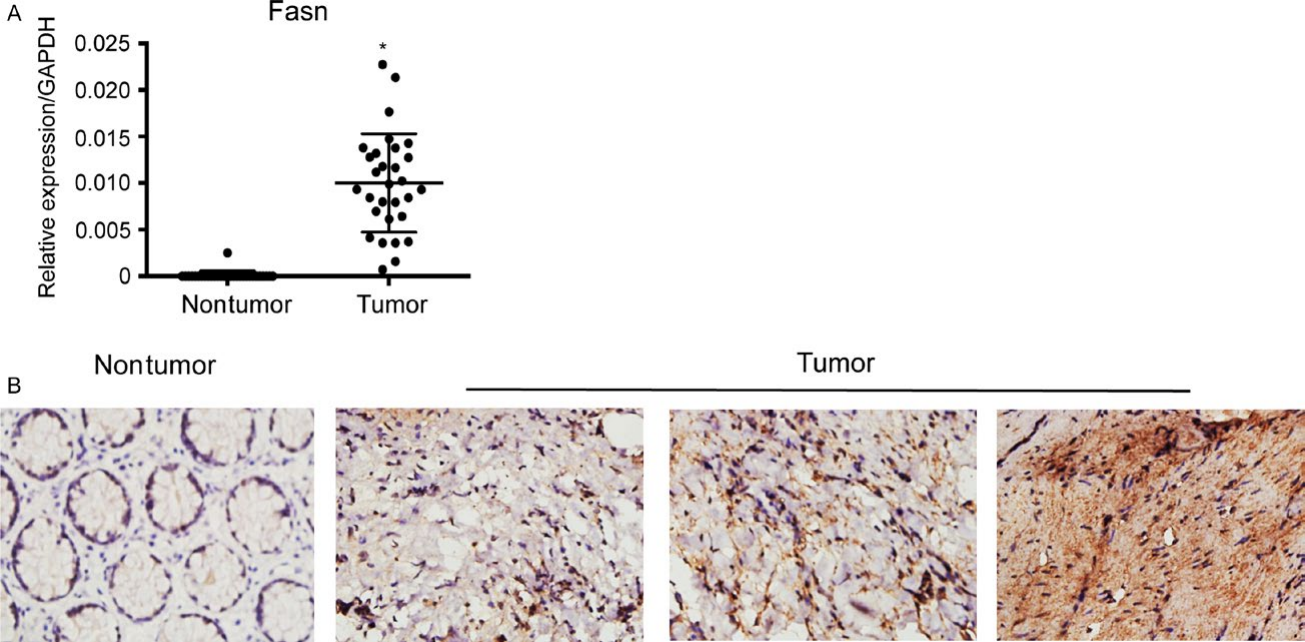
Fasn is overexpressed in CRC tissues. (A) Quantitative RT‐PCR analysis of Fasn expression in 29 pairs of colorectal tumor and its corresponding normal tissues. (B) Representative immunohistochemistry images showed the varied overexpression of Fasn in 43 CRC samples.

Fasn expression varied due to the deviation in population and geometry illustrated by previous researches. As such, we determined Fasn mRNA expression level in 29 pairs of CRC and corresponding noncancerous tissues by quantitative RT‐PCR. As shown in Figure 2A, CRC tissues exhibited remarkably elevated Fasn mRNA expression compared to adjacent normal tissues (*P* < 0.05). Further immunohistochemistry assay exhibited similar results. While in general Fasn expression is upregulated in CRC, it displayed certain extent of heterogeneity indicating certain biological function in this circumstance.

### Fasn knockdown attenuates invasion of CRC cells

**Figure 3:**
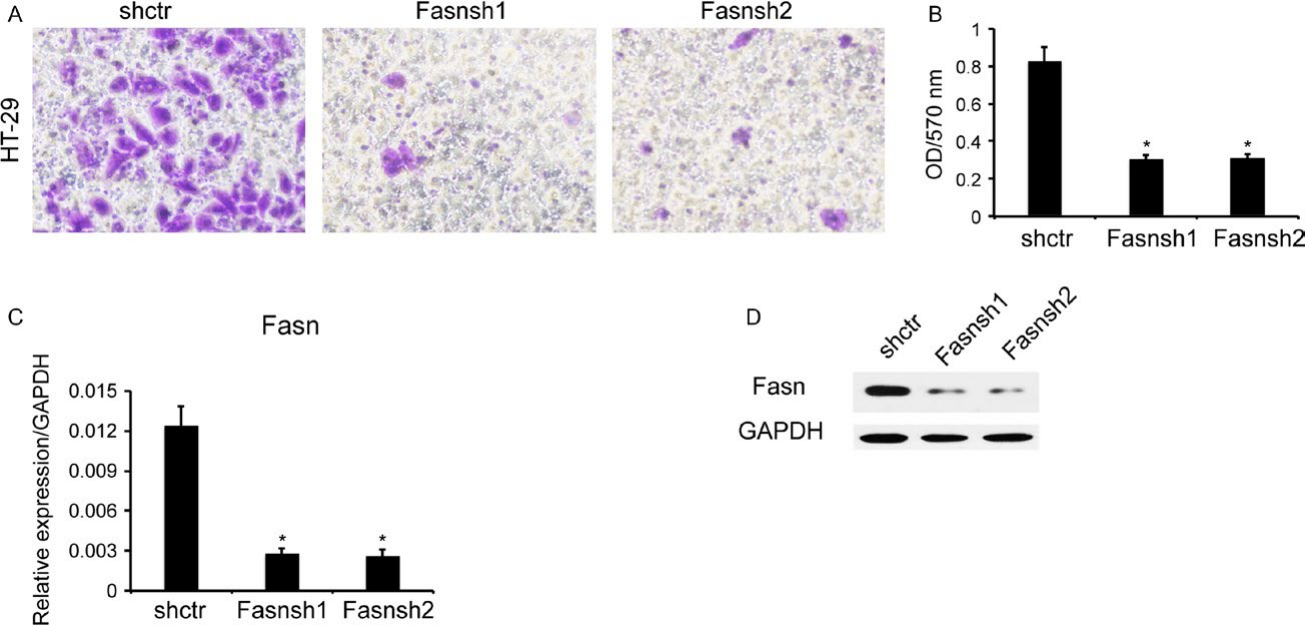
Fasn knockdown inhibited invasion in CRC cell line HT‐29. (A) Matrigel invasion assay showed that Fasn knockdown decreased invasive capacity. (B) The graph showed absorbance at 570 nm after 24 h quantifying matrigel invasion assay. *P < 0.05. (C) Quantitative RT‐PCR analysis of knockdown efficiency in Fasn knockdown stable cell lines. (D) Western blotting analysis of knockdown efficiency in Fasn knockdown stable cell lines.

**Figure 4:**
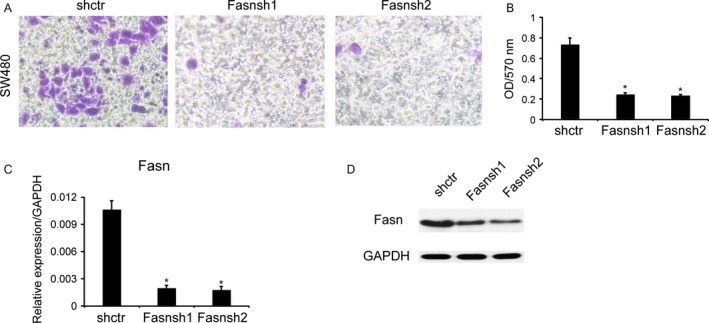
Fasn knockdown inhibited invasion in CRC cell line SW480. (A) Matrigel invasion assay showed that Fasn knockdown decreased invasive capacity. (B) The graph showed absorbance at 570 nm after 24 h quantifying matrigel invasion assay. * *P* < 0.05. (C) Quantitative RT‐PCR analysis of knockdown efficiency in Fasn knockdown stable cell lines. (D) Western blotting analysis of knockdown efficiency in Fasn knockdown stable cell lines.

Since Fasn expression is upregulated across various CRC cell lines and in CRC patients, we took on experiments to investigate its function during CRC tumorigenesis. Based on previous finding that HT‐29 and SW480 harbored relative higher expression of Fasn, we constructed Fasn knockdown stable cell line through lentiviral infection in these two cell lines. We proceeded matrigel invasion assay to analyze its take on CRC invasion and results came in showed that decreased expression of Fasn reduced invasive capabilities in HT‐29 cells (Fig. 3A and B). Fasn knockdown efficiency was confirmed on both mRNA and protein level (Fig. 3C and D). We repeated these experiments in SW480 cells and received coherent outcomes (Fig. 4), suggesting that Fasn had a unanimous pivotal role in CRC oncogenesis, that is, inducing tumor invasion in vitro.

### Fasn knockdown inhibited Wnt signaling pathway and its expression positively correlated with Wnt marker gene

**Figure 5:**
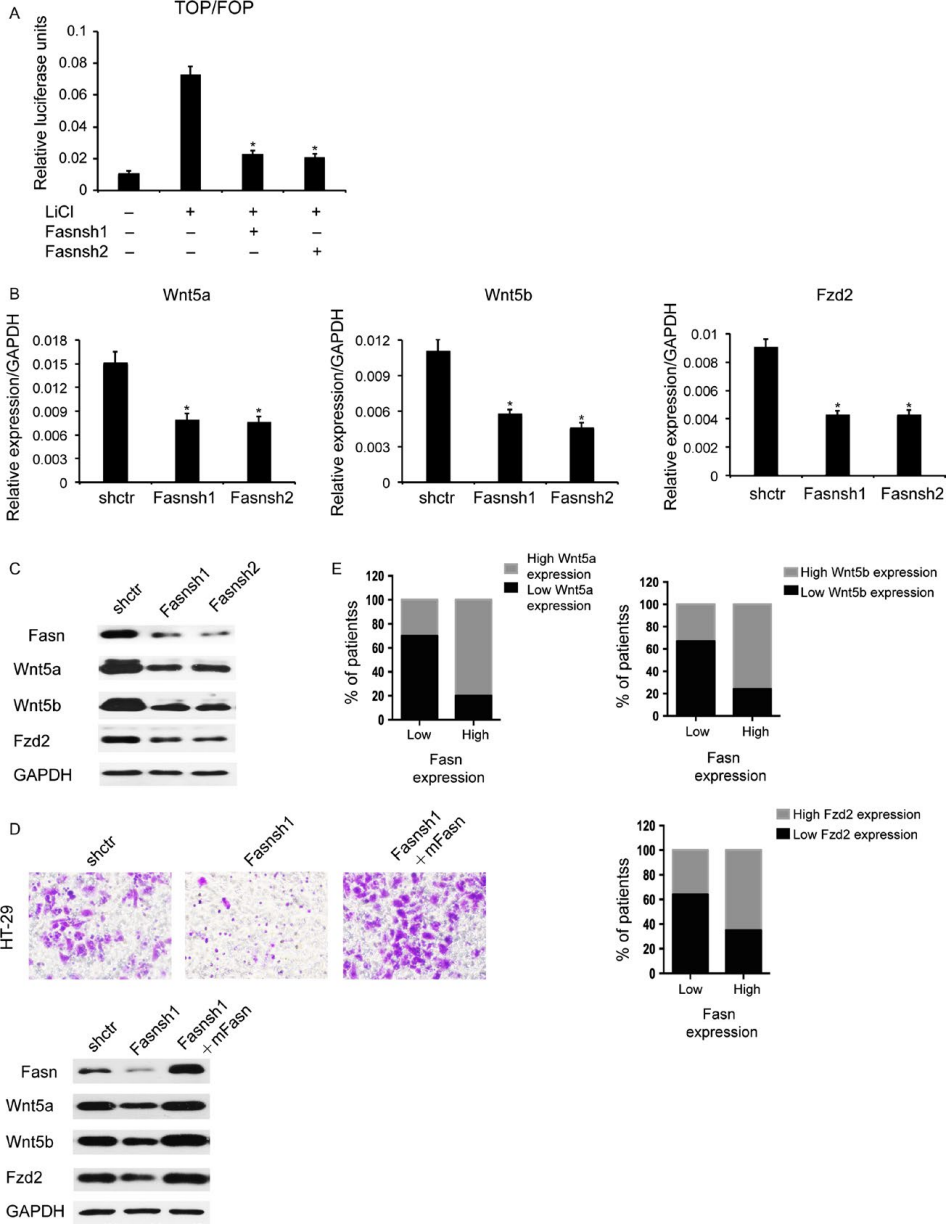
Fasn knockdown inhibited Wnt signal pathway in CRC cells and its expression positively correlated with Wnt marker genes. (A) Dual luciferase assay showed that Fasn knockdown inhibited Wnt signaling in HT‐29 cells. TOP and FOP is the promoter plasmid commonly used for Wnt signaling. (B) Quantitative RT‐PCR assay showed that Fasn knockdown reduced Wnt signaling marker gene expression in HT‐29 cells, such as Wnt5a, Wnt5b, and Fzd2. (C) Western blotting showed that Fasn knockdown reduced Wnt signaling marker genes expression in HT‐29 cells. (D) Matrigel invasion assay showed that overexpression of murine Fasn rescued human Fasn knockdown phenotype. Western blotting showed overexpression of murine Fasn increased Wnt signaling marker genes expression in HT‐29 cells. (E) Quantitative RT‐PCR analysis showed that the expression of Fasn and Wnt signaling marker gene is positively correlated in 29 pair of CRC tissues (*P < 0.05, Student's *t* test).

Tumor metastasis is a complicated process and the underlying mechanism was an enigma a decade ago, which just started to reveal 10. Recent studies have provided evidences suggesting that abnormally activated Wnt signaling pathway 11 confers on cancer cells high‐grade malignant traits 12. Therefore, we sought to determine whether Wnt participated in Fasn‐induced CRC invasion. As expected, knockdown of Fasn resulted in a drastic decrease in Wnt pathway (Fig. 5A), evidenced by downregulation of the Wnt marker genes Wnt5a, Wnt5b, and Fzd2 (Fig. 5B and C). Wnt5a, Wnt5b, and Fzd2 13 are crucial proteins reported to associate with cancer stemness and mobility. During our experiment, we also observed that compared to Fasn knockdown effectiveness the decrements in Wnt marker genes were rather delicate, indicating other signal pathways may contribute to Fasn‐enabled metastatic phenotype conjointly (Fig. 5C). For further verification, we overexpressed murine Fasn in human Fasn shRNA stable cell line (Fig. 5D) and found that overexpression of murine Fasn could reverse previous phenotype and increase Wnt5a, Wnt5b, and Fzd2 expression, thus confirming our theory. We also substantiated our findings by elaborating its connection in CRC patients. Indeed, in a cohort of 29 CRC patient samples, Fasn expression was positively correlated with Wnt5a, Wnt5b, and Fzd2 14 expression (Fig. 5E). All in all, these results showed that Fasn could emerge as important regulators of tumor aggressiveness, colligating energy metabolism, and biological functions in malignant cells, which was partly enabled by an aberrantly active Wnt signaling pathway.

### Fasn expression is negatively correlated with metastasis and prognosis in CRC

Our studies have verified higher level of Fasn in CRC samples compared to adjacent nontumorous tissues, yet its relevance with clinical outcome remained elusive. We performed tissue microarray on 43 pairs of CRC specimens and corresponding normal colorectal mucosal tissues. An additional correlation analysis unveiled that Fasn expression level was negatively correlated with CRC metastasis and advanced pathological TNM stage (Table 1). Taken together, Fasn could serve as a potential biomarker for metastatic CRC.

**Table 1:** Clinicopathological correlation of Fasn expression in human sporatic colorectal carcinoma

Feature	Fasn		*χ* ^2^	*P* value^b^
	Low	High^a^		
All cases	21	22		
Gender				
Male	13	12	0.239	0.625
Female	8	10		
Age				
>65	11	13	0.196	0.658
≤65	10	9		
Intravascular cancer embolus				
Present	2	9	5.559	0.021
Absent	19	13		
Perineuronal invasion				
Present	1	7	5.194	0.027
Absent	20	15		
Tumor stage				
I	5	1	9.809	0.018
II	8	3		
III	7	12		
IV	1	6		

a The median expression level was used as the cutoff. Low expression of Fasn in 21 patients was classified as values below the 50th percentile. High Fasn expression in 22 patients was classified as values at or above the 50th percentile.

b For analysis of correlation between Fasn and clinical features, Pearson's chi‐square tests were used. Results were considered statistically significant at *P* < 0.05.

## Discussion

Rapidly proliferating cancer cells exhibit considerably different metabolic requirements 15 to normal counterparts in order to support cell growth and survival. Metabolic pathways therefore are rewired to meet their high needs for energy and macromolecules. Among all the metabolic alterations, recent lines of evidence suggest 16 that activation of the de novo fatty acid synthesis pathway is indispensable for carcinogenesis. Fatty acids contribute to cancer progression manifold 17, including energy supply and protumorigenic signal molecules biosynthesis.

The hyperactivated de novo lipogenesis in tumor cells is reflected by a drastic increase in various lipogenic enzymes activity. Fatty acid synthase (Fasn), the major enzyme responsible for fatty acid biosynthesis, are found to correlate with poor prognosis in some cancer types 18, while in CRC patients Fasn expression on clinical outcome varied with geography and ethnic. Our findings indicate that cellular Fasn expression is upregulated in CRC patients in Asian and higher Fasn level are more frequent in advanced CRC patients. In vitro studies confirmed that knockdown Fasn in various CRC cell lines hindered invasive capability of cancer cells in accordance with our clinical analysis, both indicating a prometastatic role of Fasn in CRC tumorigenesis. Fasn represents a crucial link between nutrient synthesis and malignancy, which is open for clinical exploit.

Wnt signaling plays a critical role in embryonic development 19, which is well established, while its role in cancer was first described three decades ago 20 in Wnt1 transgenic mouse models of mammary cancer. Subsequently, more studies revealed a critical role for aberrant Wnt signaling in CRC involving diverse cellular processes as cell migration 21 and polarity and stemness 22. Wnt ligand and transmembrane receptors Fzd 23 family constitutes a fundamental part of both canonical and noncanonical Wnt signaling.

In this study, with dual luciferase report assay, quantitative PCR, and immunoblotting, we were able to elaborate that Fasn knockdown significantly inhibited Wnt signal pathway and a Wnt5a/5b‐Fzd2 transduction circuit was featured in this Fasn‐driven metastatic process. The specific underlying mechanism of this progress needs further exploration and one possible explanation is that Fasn catalyzes the synthesis of palmitate, which accounts for an important posttranslational modification of protein palmitoylation. The palmitate moiety of Wnt is crucial for signal transduction to *β*‐catenin and proper extracellular secretion. However, the effects of Fasn exerting on Wnt signaling may not limit to palmitoylation. We also found that Wnt signaling suppression extent was out of proportion to Fasn knockdown efficiency, indicating that other signal pathway may contribute to this phenotype synergizing with Wnt.

In conclusion, we demonstrated that Fasn overexpression conferred metastatic advantages on CRC cells besides functioning in an anabolic energy storage way. Moreover, Fasn inhibitors have been put into clinical use for hyperlipidemia treatment decades ago and its safety has been approved. Given that upregulation of Fasn represents a nearly universal phenotypic alteration in most human malignancies, Fasn could be conveniently applied for anti‐CRC therapy and other diverse cancer types upon further verification. This discovery holds the promise of resolving unsettled problems and yield innovative therapeutics when targeting colon cancer metabolically.

## Conflict of Interest

None declared.
